# Evaluation framework for facilitating the technology transfers of universities: Focusing on the perspective of technology donors

**DOI:** 10.1371/journal.pone.0293951

**Published:** 2023-12-14

**Authors:** Jongyi Hong, Jeonghwa Cha, Bilegjargal G., Kyungbo Park

**Affiliations:** 1 Institute for Research & Industry Cooperation, Pusan National University, Geumjeong-gu, Busan, Republic of Korea; 2 Department of Business Administration, Pusan National University, Geumjeong-gu, Busan, Republic of Korea; 3 Department of Business Administration, Andong National University, Andong, Gyeongsangbuk-do, Republic of Korea; Instituto Tecnologico Autonomo de Mexico, MEXICO

## Abstract

Technological innovation and preoccupation with new markets through technological innovation have become critical factors in achieving success in the global market. Currently, companies cannot develop and commercialize all technologies. Therefore, the importance of technology transfers is rapidly increasing. Technology transfer is a crucial strategy adopted by organizations to remain innovative and competitive. However, Korea’s technology transfer rate is only 37.9%. In particular, the technology transfer rate from universities to companies is lower than that from government-funded research institutes in Korea. Although the fundamental approach for resolving barriers to technology transfer have been studied, previous research has been conducted from a narrow definition of technology transfer. Furthermore, previous research has focused on analyzing the success factors of technology transfer, presenting technology transfer processes, or conducting case studies. Therefore, it is necessary to develop a technology donor diagnosis framework based on CSFs (Critical Success Factors) to eliminate obstacles to technology transfers. To lower the barriers to technology transfers, it is necessary to develop a strategy for a successful technology transfer based on the diagnosis of technology donors. This study develops a diagnosis framework for universities from the perspective of technology donors, implements and tests the framework using case studies, and proposes strategies for each stage of technology transfer growth. The framework is able to assess multidimensional perspectives, because CSFs and PMs were extracted based on BSC. Furthermore, by comparing the perspectives score of technology donors in different universities, technology donors can identify the areas in which each university is lacking in its current situation. Multidimensional diagnosis and aggregation score of technology donors offer to extract optimal CSFs for technology transfer activation for each growth stage.

## Section 1: Introduction

The effective acquisition and utilization of new technologies are essential aspects of corporate success and critical determinants of corporate competitiveness [1]. The radical development of technology is rapidly changing the innovation rates for both products and services [2]. A company can lead the global market by securing efficiency in a series of processes, from the start of research and development (R&D) to commercialization [3]. Technological innovation and preoccupation with new markets through technological innovation have become critical factors in achieving success in the global market. Currently, companies cannot develop and commercialize all technologies. Therefore, the importance of technology transfers is rapidly increasing [4]. Technology transfer is a crucial strategy adopted by organizations to remain innovative and competitive. The importance of technology transfer lies in its ability to promote innovation and enhance competitiveness. By transferring knowledge, expertise, and technology from one organization to another, technology transfer can facilitate the development of new products and services, improve production processes, and create new markets. Furthermore, technology transfer can help organizations stay at the forefront of their industries and adapt to changing market conditions. In this way, technology transfer plays a vital role in driving economic growth and promoting societal progress. More than 50% of new product development and service innovations occur through technology transfers [5]. The market launch of new products and services based on technology transfers provides an opportunity for a company to improve its profit and market share [6]. As the importance of technology transfers increases, technology transfers in universities likewise increases [4]. In particular, as the contribution of universities to technology transfers increases, the role of universities as technology donors also increases [7, 8].

Not only the Korean government but also government of many countries have been continuously increasing their government R&D budgets. The Ministry of Industry and Energy in Korea is formulating and promoting various policies for technology transfer and commercialization. The technology transfer rate from public research institute to company in the United States is 42.4% in 2020. However, Korea’s technology transfer rate is only 37.9%. In particular, the technology transfer rate from universities to companies is lower than that from government-funded research institutes in Korea. The commercialization and business expansion of technology produced through university R&D activities should be transferred to companies for visible results. However, in Korea, technology transfer through university R&D is hindered by barriers and is not being activated. The main reason for the hindered technology transfer activation is the existence of technology transfer barriers [9, 10]. Various studies had been conducted to identify the barriers that lead to technology transfer failures, in order to promote the facilitation of technology transfer. Although the fundamental approach for resolving barriers to technology transfer have been studied, previous research has been conducted from a narrow definition of technology transfer. Technology transfer was defined based on specific academic fields and research purposes [1]. Early research defined technology transfers between organizations [9]. Previous research focusing on technology has defined technology transfers as efficient technology transfer policies from a technology donor to a technology user. However, the definition of existing technology transfer barriers needs to be redefined due to the expansion of the definition of technology transfer, because successful technology transfer is not simply about transferring technology, but also depends on a range of factors, such as the technology user’s ability to absorb the technology and the suitability of the technology for the technology user’s needs. Therefore, evaluating technology transfer donors, considering these factors, is essential. In other words, successful technology transfers are possible if the technology donor diagnoses the technology donor based on its diagnostic framework for the activation of technology transfer and supports the technology donor in deriving the key indicators required for a successful technology transfer [10].

Most research related to technology transfer do not diagnose or evaluate technology donors but instead focuses on developing common key factors or cases to achieve success in technology transfers. Although diagnosing technology donors based on the framework of technology transfer activation is the key to a successful technology transfer [11], research on diagnosing technology transfer, analyzing technology donors, and deriving strategies is scarce. The most significant barrier to technology transfers is the absence of a technology transfer strategy caused by a lack of knowledge among technology transfer donors. The primary obstacles are the technology transfer activation strategy that the technology donor must select for a successful technology transfer and the technology donor’s choice of technology transfer type. The selection of an efficient technology donor policy is a crucial success factor in technology transfers [12]. However, previous research has focused on analyzing the success factors of technology transfer, presenting technology transfer processes, or conducting case studies. Research focusing on technology transfer evaluation has also focused on proposing technology valuation models, analyzing the efficiency and effectiveness of technology transfer, or validating technology transfer paths.

Therefore, it is necessary to develop a technology donor diagnosis framework based on CSFs (Critical Success Factors) to eliminate obstacles to technology transfers. To lower the barriers to technology transfers, it is necessary to develop a strategy for a successful technology transfer based on the diagnosis of technology donors. The objectives of this study are as follows.

■ The first objective is to develop a technology donor diagnosis framework to reduce barriers of technology transfer. The framework aims to diagnose universities, the technology donors, from various perspectives, and derive CSFs that are linked to the strategies. CSFs are derived using the process of building a BSC (Balanced Scorecard).
■ The second objective is to develop a framework for deriving the normalized score of CSF. The PMs (Performance Measures) that make up CSF have different relative importance and measurement units. To diagnose the technology donors and derive strategies based on the diagnosis, it is essential to calculate the aggregation Score for each CSF. Therefore, in this study, we aim to derive the relative importance of PMs based on AHP (Analytic Hierarchical Process) and normalize the scores of each PMs based on the normal distribution.
■ This study aims to derive strategies based on diagnosis, rather than just evaluation. To do this, the technology donors are classified according to their growth stages, and the scores of the technology donors are compared to those of others in the same growth stage to identify the CSFs that are lacking. By comparing technology donors in the same growth stage, optimized strategies for improving technology transfer will be derived.

This study develops a diagnosis framework for universities from the perspective of technology donors, implements and tests the framework using case studies, and proposes strategies for each stage of technology transfer growth. The research was conducted according to the following structure to achieve the research purpose. Section 2 presents a literature review on how technology transfers were evaluated from previous research and technology transfer perspectives. This helps determine the subsequent research methodology and achieve a basic understanding. Section 3 provides detailed research on the methods used and explains the diagnostic framework used step by step in this study. Section 4 examines the technology donor’s diagnostic framework presented in the previous chapter using a case study and summarizes the results. Section 5 compares the present study with existing research and highlights the specifics and strengths of the current investigation. Finally, Section 6 summarizes the research conclusions, provides further suggestions, details the technology donor’s diagnosis, and evaluates the research limitations.

## Section 2: Literature review

Section 2 analyzes the literature on technology transfers. The section is divided into three sub-topics. The first lays the theoretical foundations for research by collecting and consolidating previous research on technology transfer activation. The second summarizes previous research on technology transfer evaluation. The third summarizes prior research on university technology transfers.

### Research related to technology transfer activation

The research related to technology transfer activation can be classified into activation strategy research and technology transfer models. Research that focuses on activation strategies can be classified into research that analyzes the establishment of institutions or policy derivation for the activation of technology transfers and research that derives and verifies success and failure factors. Moreover, the technology transfer model can be classified into presenting the technology transfer process through theoretical analysis and demonstrating the proposed model through case research. The Table 1 presents a classification of the related literature based on the criteria presented in this study.

**Table 1:** Research related to technology transfer activation.

Classification criteria	Detailed criteria	Related literature
**Activation strategies**	Institutions and policies	[13–26]
	Success and failure factors of technology transfer	[5, 17–20, 27–34]
**Technology transfer model**	Technology transfer process	[9, 35–39, 41]
	Case research	[23, 40, 42–45, 47–52]

There have been studies not only on the aforementioned research, but also on criteria for selecting potential technology transfer partners [35–37, 41]. Criteria for selecting potential technology partners include compatibility, intellectual property, market potential, financial resources, reputation, and more.

There have also been studies on determining potential markets related to technology transfer [40–42, 47–50]. In almost all studies, after identifying the target market related to the technology to be transferred, market research is conducted to identify the strengths and weaknesses of existing products or services, technologies, and potential entry barriers. Then, considering regulatory and legal factors [39], consultation with industry stakeholders can ultimately determine the potential market related to the technology [36]. Overall, evaluating potential markets for technology transfer opportunities involves careful analysis of several key factors to ensure a strong value proposition for viable markets and potential customers. The evaluating a technology transfer opportunity requires consideration of several criteria, including market potential [40–42], intellectual property protection [47], technical feasibility [27], financial feasibility [17–19], and commercialization strategy [48–50]. By carefully evaluating these factors, technology transfer opportunities can be effectively evaluated and commercialized.

### Research related to technology transfer evaluation

There are no general theories, models, structures, or explanatory theories on technology transfer [62]. Moreover, there is currently no system for evaluating the success of technology transfers before a product is designed, manufactured, marketed, or used. From the perspective of an organization, technology tends to be evaluated only in terms of usability and functionality. However, transferring technology from one stakeholder to another requires the assessment of a broader context [53]. Until now, when evaluating technology transfer, most studies have focused on evaluating organizations that receive technology [9, 24–26, 30–32]. Therefore, the receiving organization has been studied continuously, and evaluating the receiving organization’s technical readiness has included evaluating the organization’s technical infrastructure, capabilities, and expertise to effectively receive and utilize transferred technology [31, 32]. This included the organization’s existing skills and R&D capabilities [17–19], and experience in implementing and managing similar technologies [23, 28]. In a similar sense, technology compatibility is becoming an important issue. The assessing the compatibility of the recipient organization’s business model and culture with the technology being transferred is a critical step in the technology transfer process. By evaluating the recipient organization’s business model, organizational culture, and technology infrastructure, a thorough assessment of compatibility can be made, which can help ensure the success of the technology transfer [28]. In addition, successful technology transfer was attempted by evaluating the organization’s workforce and identifying gaps in technical skills or expertise [29]. Furthermore managing potential conflicts of interest is important to ensure that technology transfers are carried out ethically and responsibly as a whole. Processes such as third-party assessment, ethical guidance, conflict of interest management planning, supervision and review can help manage potential conflicts of interest and ensure ethical and responsible technology transfer [34].

Lastly, to assess the risks associated with a technology transfer, a risk management plan should be developed that includes identifying potential risks, analyzing their impact, and developing mitigation strategies. Measures to mitigate risk may include conducting due diligence, implementing contracts and agreements, establishing quality control measures, developing contingency plans, and providing training and support to recipients. The effectiveness of risk mitigation strategies should be regularly monitored and reviewed to ensure that risks are effectively managed throughout the technology transfer process [37–39].

Technology transfers can be classified into technology and knowledge according to the scope of the evaluation target. Research that evaluates technology can be classified into research that evaluates technology value according to research methods, research that assesses the effectiveness and efficiency of technology transfer, or research that analyzes the pathways of technology transfer. Research evaluating technology has focused only on technology valuation, and there has been little research that diagnoses technology donors or suggests strategies. However, it is necessary to comprehensively assess technology-related knowledge and expertise regarding technology transfers. Furthermore, most studies have only developed technological evaluation and diagnostic methodologies. The research related to the assessment of technology transfer is detailed in Table 2 below.

**Table 2:** Technology transfer evaluation research.

Evaluation target	Evaluation method	Related Literature
**Technology**	Technology Valuation Model	[16–18, 20, 25, 32, 46, 47, 54–60, 62, 63]
	Effectiveness and Efficiency Analysis	[4, 19, 21, 61, 64, 66, 67]
	Path Analysis	[24, 33, 49, 50, 65, 68–71]

Most research has used different technology valuation models, with each model having its own scope, level of implementation, and methodology. For example, refer to the redevelopment of models based on previous research [16, 18, 32, 47, 62, 63], financial and statistical forecasting based on mathematical calculations [17, 20, 25], and the creation of a complex model using a combination of different methods [32, 47, 62]. In terms of efficiency, research was mainly conducted to estimate the efficiency of technology transfers in a particular sector [19, 21, 67]. And to use the above method, existing studies have established indicators to measure success before technology. These indicators include intellectual property creation, revenue generation, cost savings achieved, developed products or services, patent applications or permits, job creation, and entry into new markets [16, 17]. Regular evaluation of these metrics can help determine the success of technology transfer and identify areas to improve in future technology transfer initiatives. Therefore, this study will also explore the indicators used in the previous study to establish the most influential indicators.

### Research related to technology transfer evaluation at the university

Modern entrepreneurial universities play a role in changing today’s competitive society [73]. Higher education research suggests that universities are "the birthplace of a wide range of disciplines, with a detailed understanding of the types of problems faced by marginalized communities and the opportunities to address them" [74]. Universities play an important role in the technology transfer process. Universities are primarily responsible for innovative research and development and can convert them into commercially available products and services [73]. Through partnerships and collaborations with industry, universities reduce the gap between research and commercialization and help innovative technologies reach the marketplace and generate social benefits [73–75]. As part of this perspective, there is a trend in university technology transfer research that identifies the ways universities can contribute to socioeconomic development through innovation [75–82]. Technology transfers are a form of commercializing university research through the influence of knowledge, public participation and innovation [83].

Recently, universities have added new dimensions to their core research and training goals, with the additional purpose of commercializing their research-based technology. This dimension is subject to a broad field of research called technology transfers. It is a process by which universities use their dynamic capabilities to recognize and respond to changing opportunities and challenges [20]. Universities have emphasized the entrepreneurial nature of technology transfers, established start-up businesses engaging many professors and students, and developed fully fledged business ecosystems such as incubators [26]. In the official form of University Technology Transfers, universities protect the research results in the condition of intellectual property, which is then "sold" to factories or end-users. Profits from this activity are believed to have been reinvested into universities to fund further research [16].

The Table 3 below presents a literature review of research related to technology transfers at universities, which is the subject of this study. Research related to technology transfers at universities can be divided into research on technology transfers between universities and companies, technology transfers between universities and colleges, and venture startups within universities [72]. Technology transfers between universities and companies is the most frequent type, whereas the commercialization rate of technology transfers is the lowest [84]. University technology transfer-related research does not evaluate university technology transfers from a comprehensive perspective but instead analyzes technology transfer performance from a specific point of view. More research is needed to identify university technology transfers from a broader perspective and derive strategies based on it.

**Table 3:** University-enterprise technology transfer-related research.

Evaluation perspective	Related literature
Cognitive aspects	[16, 85–94]
Geographical aspects	[52, 85–91, 93–101]
Organizational aspects	[23, 33, 85–91, 93–97, 102]
Social aspects	[20, 24, 85–91; 93, 94, 97, 100, 102]

Many types of research are related to university technology transfers. The latest research trends include university research examining the efficiency of technology transfers to companies, research on the implementation of activities between countries and universities, and research investigating the transfer of nonprofit technology for the benefit of society. Furthermore, the implementation and role of the technology transfer department within the university [23, 24], staff skills issues [26, 33], and research based on university technology transfer implementation organizations have been conducted. Moreover, historical research on the growth and development of university technology transfer [52], its impact on academic entrepreneurship [16, 20], and the internal and external impacts of economic change on university technology transfer [20] has been conducted.

University-specific factors have been examined in the transfer of technological, human, and financial resources for research and technology licensing [105–108], and their effects on technology experience transfer \ [31, 109, 110]. Studies investigating administrative structures to support research and commercial activities (technology transfer offices, research departments, incubators) [111, 112], forms of ownership (private, public, or mixed) [27, 113], and formal and informal university institutions related to technology transfers [114] have shown effects on university technology transfer results. Research on university technology transfers suggests that there is a conflict between the scientific norm of entrepreneurship in the research sector, the rapid dissemination of research results, and the commercialization of research, which may represent a significant obstacle to university technology transfers [115–117]. Most research universities have a business ecosystem with property-based organizations, such as technology transfers and accelerators, which can stimulate entrepreneurship, and science/technology/research parks [118–120].

## Section 3: Research framework

**Fig 1:**
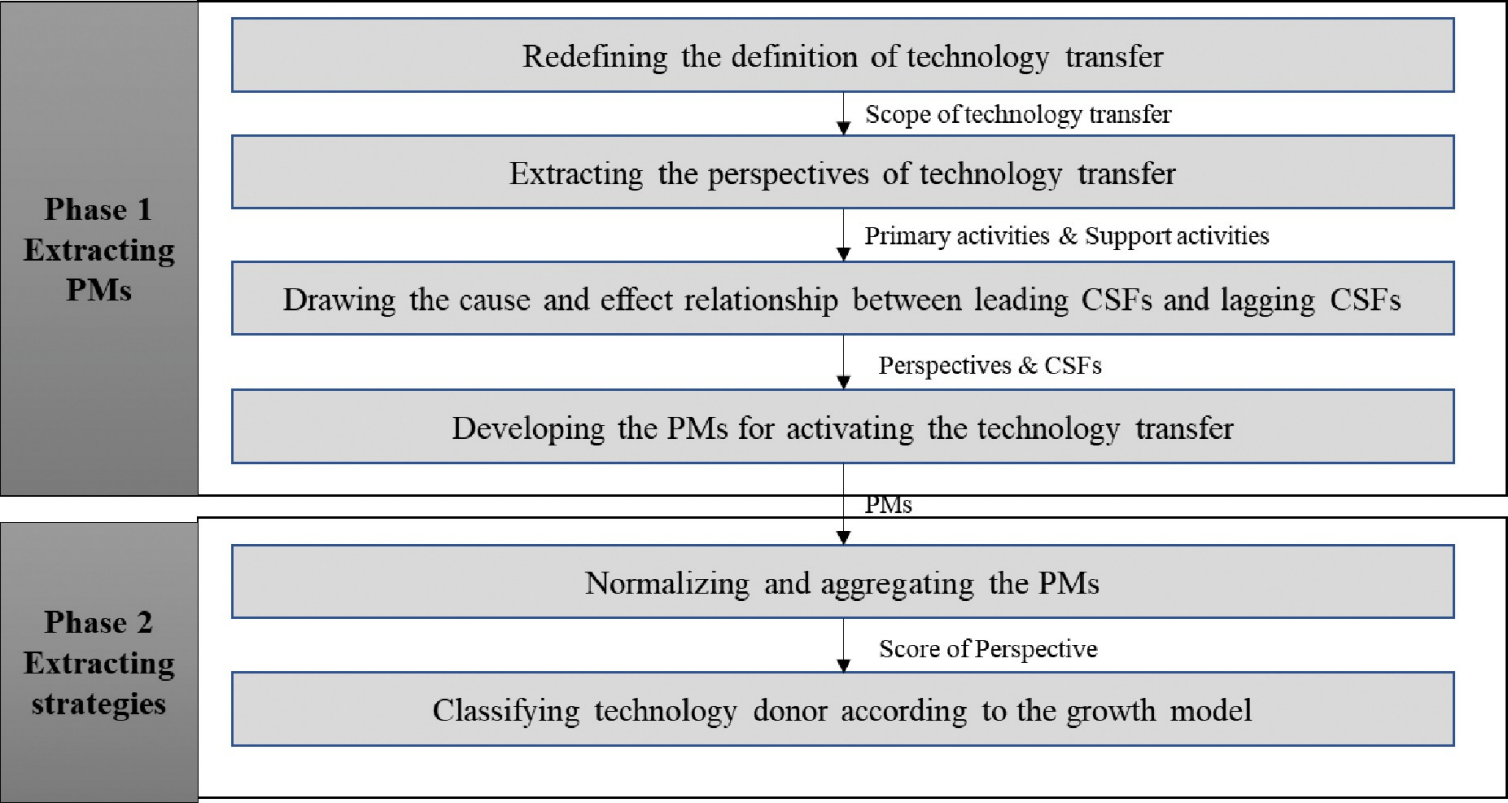
Technology donor diagnosis framework.

The research methodology is illustrated in the following Fig 1. The technology donor diagnosis framework comprises two main phases.

The first phase comprises the PMs (Performance Measures) extraction method and a cause-and-effect diagram to reveal the causal relationship between CSFs (Critical Success Factors). Most previous research related to technology transfers between technology donors or universities measured single aspects of technology transfers, such as organizational or engineering aspects. To evaluate the technology donor to be linked to the process rather than a simple measurement, the PM should be drawn from the technology transfer strategy and the CSF should be connected [121]. Therefore, the cause-and-effect relationship is used to derive perspectives, strategies, and CSF.

As the PMs derived from Phase 1 have different measurement units, a normalization method should be used. Because the weights of each PM are also different and the PM has a causal relationship, an Analytic Network Process (ANP) should be used. It is possible to calculate the normalized score by point of view through the normalization and aggregation methods. Thus, it is possible to establish a strategy for each university (technology donor).

### Phase 1: Development of critical indicators for technology donor diagnosis

#### Redefining the process of technology transfers

Technology transfers have been described in various ways in research and academic studies [122]. In initial research, technology transfer flow was defined as the flow of technology from a technician donor to a technology recipient. Intellectual property rights or inventions move from academic research to industry through licenses (use right) [122]. The process of technology transfers involves transferring a design to any organization for commercialization under a license contract between other organizations (or individuals) [123]. It also involves the transfer of technical knowledge and research results from universities to potential users. The definitions used in most studies have focused on a one-way process to deliver technology from technology donors to technology recipients. However, the commercialization of university-owned intellectual property, known as university technology transfers, is a complex process in which universities attempt to identify, patent, and license professors’ inventions. These activities significantly impact the economy, resulting in a wide range of studies focusing on the interpretation, explanation, and improvement of technology transfer processes [124–127]. Therefore, this study redefines technology transfer by comparing and summarizing the theories of 35 research papers related to technology transfers published between 2015 and 2022.

#### Extracting the perspectives of technology transfers

The Korean government’s R&D budget has continuously increased over the last several years. The Korean government and major science and technology countries are constantly expanding their R&D budgets. The Ministry of Trade, Industry, and Energy has established and promoted various technology transfer and commercialization policies. However, the technology transfer rates of the significant countries in science and technology are 35.9% in the United States and 46.7% in Europe, whereas in South Korea, they are only 24.2% [103].

Despite the increasing outcomes of university technology commercialization, evidence shows that TTOs (Technology Transfer Organizations) need to enhance their efficiency. For instance, although licensing revenue rose from 48,320 million Korean won in 2011 (50,887 million at the 2015 constant price) to 68,489 million Korean won in 2015, TTOs and universities paid 57,119 million won in registration and maintenance fees in 2015. Furthermore, the licensing revenue of universities still indicates a low leverage of government R&D spending in South Korea at only 1.41% in 2018. The average contribution of licensing revenue to the overall university revenue was approximately 0.9% in 2016. Considering this situation, researchers, the government, and university administrators in South Korea have begun questioning the role of TTOs in developing innovation and knowledge-based economies [129, 130].

The results are only tangible when the technology produced through the R&D activities of the university is commercialized through transfer to the company. However, in Korea, technology developed through university R&D is blocked by technology transfer barriers, which prevent the activation of technology transfer [104]. There are several barriers for the inhibition of the activation of technology transfer. However, due to the expansion of the meaning of technology transfer, the existing definition of barriers to technology transfer must be redefined. Technology transfer barriers were redefined based on the literature review. The first barrier to technology transfer is the absence of technology transfer strategies of technology donors [104, 128, 131]. Even after market research on necessary technologies or technology applications, technology transfers fail because the technology donor fails to properly select the type and method of technology transfer. Further, technology users need knowledge not only about technology but also about technology application processes, technology innovation processes, and technology utilization-related knowledge (know-how, best practice). This is because technology transfers cannot be achieved directly by transferring technology licenses or intellectual property rights. The second barrier is a lack of information regarding the members of the technology donor [2, 128, 131]. To act, it is necessary to transfer knowledge rather than directly transfer technology to activate technology transfer cases, and implicit learning is more important than explicit knowledge [5] for the transfer of tacit knowledge, it is necessary to identify the members of the technology donor for the transferred technology. However, technology transfers fail because of the recruitment and forced participation of inappropriate personnel for technology transfer. The third barrier is the institutions related to technology transfers [2, 3, 104, 131, 132] Most of the research related to barriers to technology transfers verifies that the main factors hindering technology transfers are the system and the regulations for non-financial and technology transfers. The final barrier is the cultural and environmental differences between providers and technology users [2, 3, 104, 131–133].

#### Derivation of CSF for technology donor diagnosis

In attempts to determine the pillars of technology transfers, researchers have identified many important factors that determine commercial success, such as designer involvement [138–140], proper coordination of governance mechanisms [141, 142], and the strength of the university’s technology transfer office [66, 143, 144]. However, previous studies have lacked a comprehensive survey of technology transfer factors. Therefore, this step aims to be more realistic and accurate by identifying CSFs and mapping interrelationships based on cause-and-effect relationships. This step determines CSFs using the balanced scorecard method while considering the four perspectives derived from the previous stage. The identified factors were categorized as leading and lagging CSFs, and the corresponding relationships were drawn. A causal relationship will be derived between CSFs using the cause-and-effect diagram used in the BSC (Balanced ScoreCard) and CSFs unrelated to the technology transfer value will be excluded.

Rockart [134] defined CSF as "a limited number of components that ensure the organization’s ability to compete successfully if the results are satisfactory." They developed the "CSFs method", which is intended to help executives understand what factors are essential and can create a potential competitive advantage [134, 145, 146]. Boynton and Zmud [135] defined the CSF as "going well to ensure the success of the organization" [147]. Technology transfers as a dependent variable depends on various factors [148, 149]. For a successful technology transfer process, it is important to identify these factors and the risks posed by their adverse effects must be mitigated using appropriate mechanisms. However, the transfer process will fail if these factors are not considered sufficiently and organizations will suffer substantial financial and non-financial damage [150, 151].

The literature introduces these factors as CSFs in technology transfers [152]. Parmenter [153] identified CSFs as "a list of issues or aspects of an organization’s operations that can be used to assess an organization’s health and well-being" [153]. It is essential to identify critical factors for successful technology transfers [150, 154]. However, the identified success factors are not equally important. Therefore, deciding which factor is more critical and requires more attention and concentration always poses a question for organizations [151, 155]. A multifaceted decision-making approach considers the importance and impact of different dimensions, and prioritizes each critical success factor for decision makers. The successful implementation of technology transfer is vital for technologists and managers, and it is necessary to identify and manage essential CSFs. Therefore, CSFs are essential for success [156].

The BSC has been hailed as a widely used, high-impact management tool [157], and it has been widely studied in a wide range of research and industries [158]. It was developed to address the changing competitive environment in which intangible assets and nonfinancial indicators are becoming increasingly crucial in investor decision-making [159–165]. The BSC method enables providers to translate their technology transfer into a set of CSFs and assess the technology transfer through a comprehensive set of measures, referred to as PM. The relationship between the perspectives identified through the above methodology and CSFs was drawn using the cause-and-effect diagram and confirmed by case studies. The importance of cause and effect relationship in BSC lies in its ability to provide a clear understanding of the impact that various business activities have on each other and on the overall performance of the technology donor. By establishing a cause and effect relationship between CSFs of the balanced scorecard, technology donors can identify which areas of their operations are contributing to success and which areas require improvement. This information can then be used to make informed decisions, set goals, and allocate resources in a way that maximizes the technology donor’s performance and achieves its strategic objectives. In short, cause and effect relationships help businesses to create a more cohesive and effective strategy by providing a clear understanding of how different components of the BSC relate to one another. Using a cause and effect diagram, technology donors can not only verify the relationships between CSFs but also derive CSFs for achieving the ultimate vision [160, 161]. The relationship between the perspectives identified through the above methodology and CSFs was drawn using a cause-and-effect diagram, a tool that offers several advantages in this context. The simplicity and visual clarity of the cause-and-effect diagram allow for an easy understanding of complex processes, simplifying the identification and presentation of potential causes related to our identified CSFs [159]. Moreover, this type of diagram encourages comprehensive thinking when analyzing problems, aiding in uncovering underlying issues that may not be immediately apparent [163]. Perhaps most importantly, it aids in identifying the root causes behind an effect or problem rather than merely addressing symptoms [162]. This effective visualization tool can lead to more insightful solutions and strategies related to our CSFs. After employing these benefits of cause-and-effect diagrams to establish relationships between various perspectives and CSFs, we further confirmed these relationships through case studies.

#### Development of PMs for diagnosing technology donors

Most previous research on technology transfer performance focused on the internal factors of an organization. According to Carlsson and Fridh [166], the transparency of inventions, research costs, and age of the TTO positively affect university patents and licenses. Some studies have found that researcher quality, R&D funding levels, TTO size, age, and the early start of technology transfer programs have a positive effect on technology transfer performance [140, 167–169]. Other researchers have used incentive mechanisms as a theoretical basis. Friedman and Silberman [27] believe that rewarding researchers, such as university location, specific mission to transfer technology, and previous experience in technology transfer positively affect technology transfer performance. Other studies have shown similar effects of the royalty rate or teacher bonus system on university license revenues [30, 142, 170–173]. Adams et al. [174] concluded that R&D collaboration agreements between public research institutes and companies promote R&D and patenting, which are beneficial for production and associated with improved technology transfer performance. Park et al. [175] examined the membership of a research consortium of public research institutes and firms and found that they increased technology transfer performance.

Previous research on technology transfer performance has focused on the role of the government as a source of research funding, ignoring the information channel [168, 169, 176]. To support technology transfers between universities and industries, the government seeks to address research inequalities and funding for R&D cooperation in the manufacturing sector [75]. Information inequality leads to both high transaction costs and inefficiencies [66, 177].

At a university, performance measurement is a structured process in which the technology transfer office identifies, measures, and monitors essential programs, systems, and procedures [178]. The technology transfer strategy revolves around the commercial functions of the technology transfer office and is related to the university’s overall strategy. After a goal is set, the actual definition of performance metrics and measurements can be initiated [179]. Developing performance metrics for various sub streams and stakeholders is becoming increasingly difficult, with complexity growing as the scope of performance becomes more diverse [180–185].

There are limitations to assessing the technology transfer of technology donors using the PMs used in specific studies. Therefore, key performance indicators for measuring CSF were derived based on a literature review. Two to three PMs were extracted to measure the CSF based on a cause-and-effect relationship analysis.

### Phase 2: Development of strategies for each growth stage of technology donors

#### Normalizing and aggregating the PMs

PMs were expressed in different quantities. Therefore, it was necessary to use normalization and aggregation methods. Based on the following formula, the PMs can be normalized:

$$p_{ij}^{n}=\int \frac{1}{\sigma_{ij}\sqrt{2\pi }}Exp{\left[-\frac{{(p}_{ij}-\mu_{ij})}{{{2\sigma }_{ij}}^{2}}\right]}^{2}$$ (1)

$p_{ij}^{n}$: Normalized value of j th PM of i th perspective

*p_ij_*: Value of j th PM of i th perspective

*σ_ij_*: Standard deviation of j th PM of i th perspective

*μ_ij_*: Mean of j th PM of i th perspective

The normalized value of the ith perspective is expressed as follows and aggregated using the following equation:

$$p_{i}^{n}=\sum p_{ij}^{n}\times W_{ij}$$ (2)

$p_{i}^{n}$: Normalized value of i th perspective

*W_ij_*: Relative importance weight of i th perspective

The relative importance weight was derived using the Analytical Hierarchy Process (AHP). The existing technology transfer evaluation-related studies have been conducted from a specific perspective. Because the scope for evaluating technology transfers is vast, there is a limit to the extent to which the focus of research can be scattered when considering all factors. Therefore, it is needed to extract and test the elements that influence the success of technology transfers. In this study, the CSF that directly affects the success of technology transfer was derived from the cause-and-effect diagram of the BSC. Subjective influence was identified by deriving the relative importance of CSF through the AHP. Moreover, an aggregation method was developed and a method for quantitative evaluation is presented. Multi-criteria decision-making (MCDM) is a popular decision-making method. It is a branch of operational research that addresses the multi-criteria decision-making process [186]. AHP has been found to be the most suitable method for solving complex decision-making problems [187–189] and is helpful for hierarchical decision-making. Therefore, it has been used in various project management decisions, including contractor selection [57], project selection [136], supplier selection [191], performance evaluation [192], stakeholder evaluation [193], quality improvement [194], and risk ranking [195–197].

#### Classifying technology donors according to technology growth model

The growth stages of technology donors are classified as follows:

■ Infant stage: Technology donors who scored low in all perspectives
■ Growth stage: Technology donors who scored high only in institutional or organizational perspectives
■ Expansion stage: Technology donors with high scores in terms of institution and organizations, and high scores in terms of human or knowledge perspectives
■ Maturity stage: Technology donors who scored high in all perspectives except for value perspective

Based on the technology donor’s diagnostic system, a strategy was proposed for each growth stage. The strategy of technology transfers, according to a technology donor’s growth stage, substantially affects the success of technology transfer. It is possible to develop a strategy based on accurate evaluation and diagnosis of technology donors. Moreover, the growth stages of technology donors were classified into the Infant, Growth, Expansion, and Maturity stages. The stage to which the technology donor belongs and the key success factors lacking among the CSFs of technology transfer are expected to lay the foundation for successful technology transfers. Therefore, we intend to develop a technology-donor diagnostic framework that includes this series of processes.

## Section 4: Case study

### Phase 1: Development of critical indicators for technology donor diagnosis

#### Redefining the process of technology transfers

This research redefines the technology transfer process by comparing and summarizing the theories of 35 research papers related to technology transfers published between 2015 and 2022.

As shown in Table 4, extensive research has been conducted on technology transfers in recent years, and they have come up with a wide range of definitions related to technology transfers. By comparing and summarizing these definitions, we redefine the definition of technology transfer in this study: “Technology transfer is the two-way technology transfer process containing information about techniques, know-how, best practices, and technology knowledge.”

**Table 4:** Definition of technology transfer by related research.

Definition of TT	Author
The one-way process of adopting foreign technology	[19, 199]
All the activities underpin the transition of a group of factors (such as knowledge, technology, and methods) from scientific research to markets	[200–202]
An active process in which advanced technologies are transferred between two different factors	[49]
Profitable operation of inter-branch technology transfer	[25, 41, 48, 53]
The development process to create innovation	[17, 18, 203]
The process of creating and trading rights through a patent	[23, 204]
The process of creating and trading rights through patents using the university’s reputation	[24, 52]
The process of entering the economy through technology transfer specialists	[26, 51]
The process of implementing technology through projects and programs	[32, 50]
The process of integrating university research into markets.	[16, 20, 127, 205, 206]
The process of introducing new equipment and know-how	[62]
The process of introducing technology through a business model	[47, 63]
The process of trading technology through the university’s technology transfer office	[22, 33, 34, 129, 207]
The two-way process of delivering technology to an organization through a technology transfer office	[21]

#### Extracting the perspectives of technology transfers

Based on the technology transfer barriers mentioned in previous studies, the perspectives on the technology transfer process can be divided into four main categories. Human factors include professionals, participants, intermediaries, and engagement in technology transfers, which are categorized into a single group. Knowledge factors are grouped according to knowledge, information, and species regarding the technology being transferred. Institutional factors that indicate involvement in the intermediary institutional or technology transfer process, are in another group. Finally, organizational factors, which refer to the receiver or transmitter of the technology, are in one group. This section illustrates these factors more clearly based on previous research.

**Fig 2:**
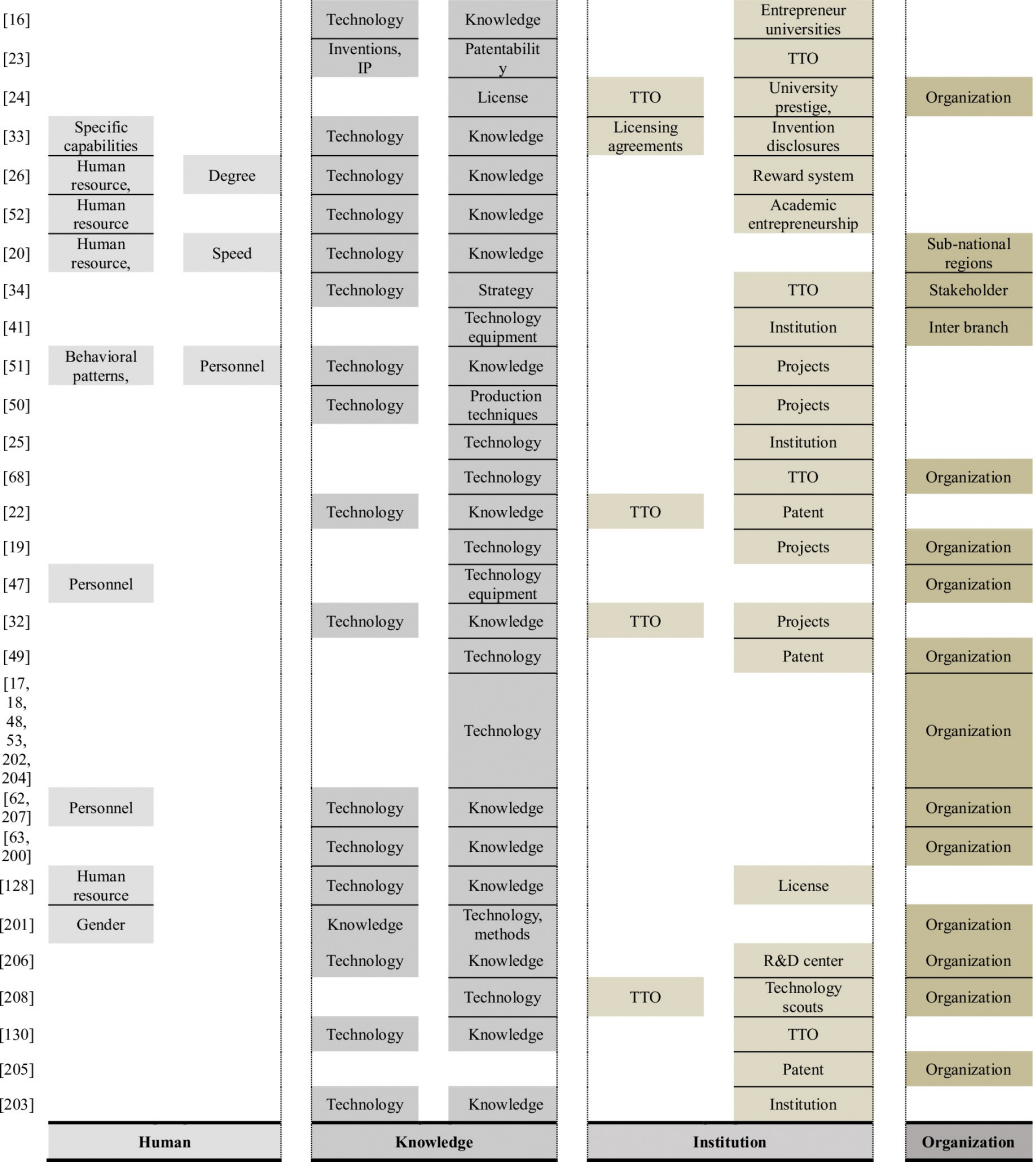
Derivation of the technology donor diagnosis point of view.

As shown in Fig 2, technology transfer providers have four central derivations. First, human perspectives include personnel, human resources, specific capabilities, behavioral patterns, education degree, gender, and speed. Knowledge perspectives include technology, network, knowledge, sharing, inventions, IP, patentability, licensing, strategy, equipment, production techniques, and methods. Third, institutional perspectives include institutions, TTO, finance, incentives, reward systems, license agreements, entrepreneurial universities, university prestige, invention disclosures, reward systems, academic entrepreneurship, projects, patents, licenses, R&D centers, and technology scouts. Finally, organizational perspectives include language and procurement, corporate culture, organizations, subnational regions, stakeholders, and interbranches.

#### Derivation of CSF for technology donor diagnosis

**Fig 3:**
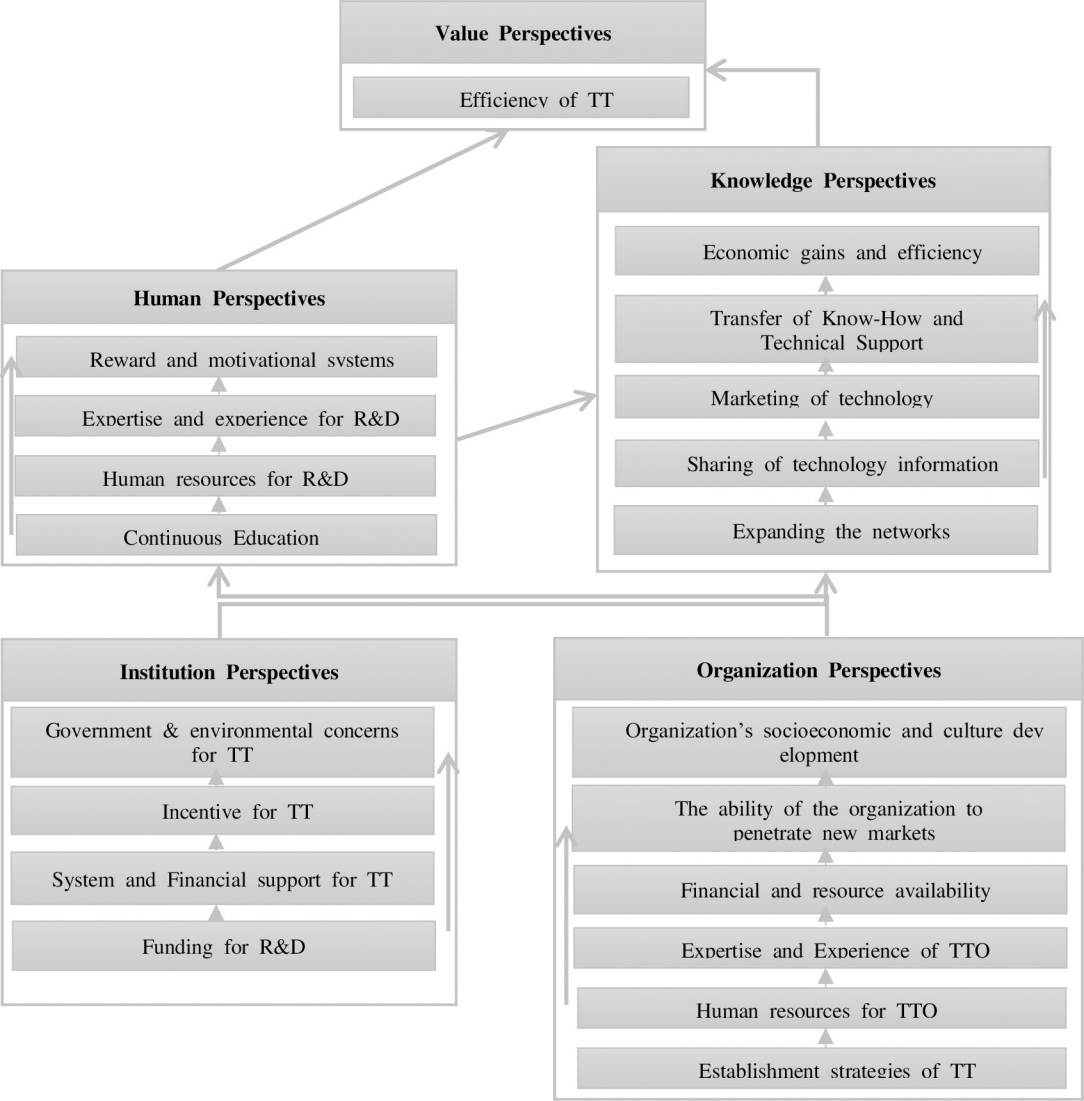
Derivation of the technology donor diagnosis perspectives.

These five perspectives with 20 CSFs were sorted from the literature review, and the cause-effect diagrams between the identified perspectives and CSFs were illustrated. In the cause-and-effect relationship diagram, the CSFs of technology transfer are categorized as leading and lagging factors, and the relationships are presented in ascending order. The cause-and-effect relationship between the leading and lagging CSFs is depicted in Fig 3.

Human and knowledge perspectives are directly related to the efficiency of technology transfer and are influenced by organizational and institutional perspectives. In the cause-and-effect relationship diagram, the CSFs of technology transfer are categorized as leading factors, and the lagging factors and relationships are illustrated in ascending order. For example, the CSFs in a human perspective, CSFs provide training, which is a form of continuous education that builds human resources for research and development. Expertise and experience in R&D are, in turn, used to create rewards and motivational systems. These cause-and-effect diagrams were meticulously crafted based on an extensive review of existing literature [66, 138–142, 208–239] and universal understanding and our perspectives. It able to derivate the technology donor diagnosis point.

#### Development of PMs (PM) for diagnosing technology donors

In this step, candidate PMs were developed to measure CSF and technology donors by designating indices that are highly relevant to CSF performance among the candidate PMs as core PMs. The Table 5 presents measurement of technology donors.

**Table 5:** Measurements of technology donors by designating indexes.

Perspectives	Strategies	CSFs	PMs
**Organization**	Strategic approach and Goal for TT [208, 209]	Organization’s socioeconomic and cultural development [63, 210, 211]	Level of sharing the vision
		The ability of the organization to penetrate new markets [203, 210, 212]	Level of sharing the goal
		Financial and resource availability [63, 155, 211, 212]	
		Establishment strategies of TT [63, 154, 155, 203, 210, 212, 213]	Level of sharing the strategy
			Level of differentiation strategy
	The outstanding capability of TTP	Expertise and Experience of TTO [154, 210, 212, 214–220]	Experience periods of TTO
		Human resources for TTO [63, 203, 210, 215, 216, 218, 220–222]	The number of members in TTO
		Expertise and experience of TTO member [212, 217, 225]	Average experience of mangers
**Institution**	Institutional support for R&D [10, 223, 224, 226, 254]	Government and environmental concerns for TT [4, 147, 203, 210]	Level of R&D guideline
		Funding for R&D [4, 147, 210, 215, 218, 220–222]	Presence of R&D funding
	Institutional support for TT [60, 213]	System and Financial support for TT [147, 154, 193, 203, 210, 212, 216, 217, 226, 227]	Management and activity cost for TT
			Protection cost
			Level of TTO support
	The incentive of technology donors [215, 218, 228]	Incentive for TT [4, 60, 147, 217, 228, 229–232]	Incentive for researcher
			Incentive for manager
**Human**	The expertise of the R&D researcher	Human resources for R&D [10, 154, 155, 210, 212, 215, 221, 224, 231]	The number of researchers
		Expertise and experience in R&D [145, 154, 210, 212, 225]	The number of PhD
	Excellent capability of member [10, 213, 232]	Continuous Education [10, 155, 208–210, 212, 227, 232]	The number of education to researcher
			The number of educations to manager.
**Knowledge**	Sharing information [234, 235]	Economic gains and efficiency in the organization	Level of post-support
		Efficient management and Sharing of technology information in organizations [63, 203, 208, 210, 212, 220, 230]	Level of DB
			External access of DB for search
		Marketing of technology in Organization [63, 209, 210, 213, 220, 236, 237]	The number of exhibition participations and advertisements
	Sharing knowledge [238]	Transfer of Know-How and Technical Support [154, 155, 203, 210, 212, 220, 236]	The cost of participations in technical presentations and advisory
	Expanding network [10, 214, 217, 239]	External Network [154, 203, 209, 212]	The number of task agreement with external agencies
			The number of external agencies
		Internal Network [155, 203, 212]	The number of regular relationships meeting between researcher and manager
**Value**	The efficiency of TT	The efficiency of TT [221]	Royalty/R&D budget
			Royalty/Researchers
			The number of TT/R&D budget
			The number of TT/Researchers

With previous research establishing a foundation, technology transfer strategies were studied, the CSFs influencing them were identified, and PMs were extracted based on CSFs.

### Phase 2: Development of strategies for each growth stage of technology donors

#### Normalizing and aggregating the PMs

All PMs were measured according to standardization and normalization formulas. The relative importance weights derived through the AHP and the statistics of the PMs are shown in the Table 6 below.

**Table 6:** Relative importance weight and statistics of PMs.

Perspectives	PMs	Relative importance weight	Min.	Max.	Average	Standard Deviation
**Organization**	Level of sharing the vision	0.098976	1.0	2.0	1.4	0.5
	Level of sharing the goal	0.059727	1.0	2.0	1.4	0.5
	Level of sharing the strategy	0.049488	1.0	2.0	1.6	0.5
	Level of differentiation strategy	0.041809	1.0	2.0	1.5	0.5
	Experience periods of TTO	0.347938	4.0	20.0	9.4	3.0
	The number of members in TTO	0.162371	0.0	30.0	3.2	5.7
	Average experience of mangers	0.239691	0.0	28.0	4.6	6.4
**Institution**	Level of R&D guideline	0.074209	1.0	2.0	1.0	0.2
	Presence of R&D funding	0.222628	1.0	2.0	1.2	0.4
	Management and activity cost for TT	0.324088	3.8	300.7	57.3	48.9
	Protection cost	0.108029	11.2	6999.0	644.7	1116.2
	Level of TTO support	0.108029	1.0	2.0	1.4	0.5
	Incentive for researcher	0.081509	2.0	3.0	2.5	0.5
	Incentive for manager	0.081509	3.0	6.0	4.5	1.1
**Human**	The number of researchers	0.5625	15.0	7566.0	780.6	1275.9
	The number of PhD	0.1875	4.0	4436.0	382.7	629.5
	The number of education to researcher	0.1875	0.1	25.0	4.6	4.7
	The number of educations to manager.	0.0625	0.3	10.0	3.5	2.3
**Knowledge**	Level of post-support	0.05861	1.0	3.0	1.5	0.6
	Level of DB	0.05861	1.0	2.0	1.4	0.5
	External access of DB for search	0.160356	1.0	2.0	1.5	0.5
	The number of exhibition participations and advertisements	0.262571	0.0	24.0	3.1	4.2
	The cost of participations in technical presentations and advisory	0.163017	0.0	500.0	10.0	47.8
	The number of task agreement with external agencies	0.059367	2.0	5.0	3.3	0.9
	The number of external agencies	0.178102	0.0	24.0	1.8	3.4
	The number of regular relationships meeting between researcher and manager	0.059367	0.0	100.0	2.9	10.0
**Value**	Royalty/R&D budget	0.123762	0.0	0.1	0.0	0.0
	Royalty/Researchers	0.222772	0.0	3.6	0.5	0.7
	The number of TT/R&D budget	0.163366	0.0	0.0	0.0	0.0
	The number of TT/Researchers	0.490099	0.0	1.1	0.1	0.1

#### Classifying technology donors according to technology growth model

The following Table 7 were obtained by classifying 118 universities according to their growth stages.

**Table 7:** Classification of technology transfer donors into growth stages.

Infant stage	Growth stage	Expansion stage	Maturity stage
37	34	31	15

**Fig 4:**
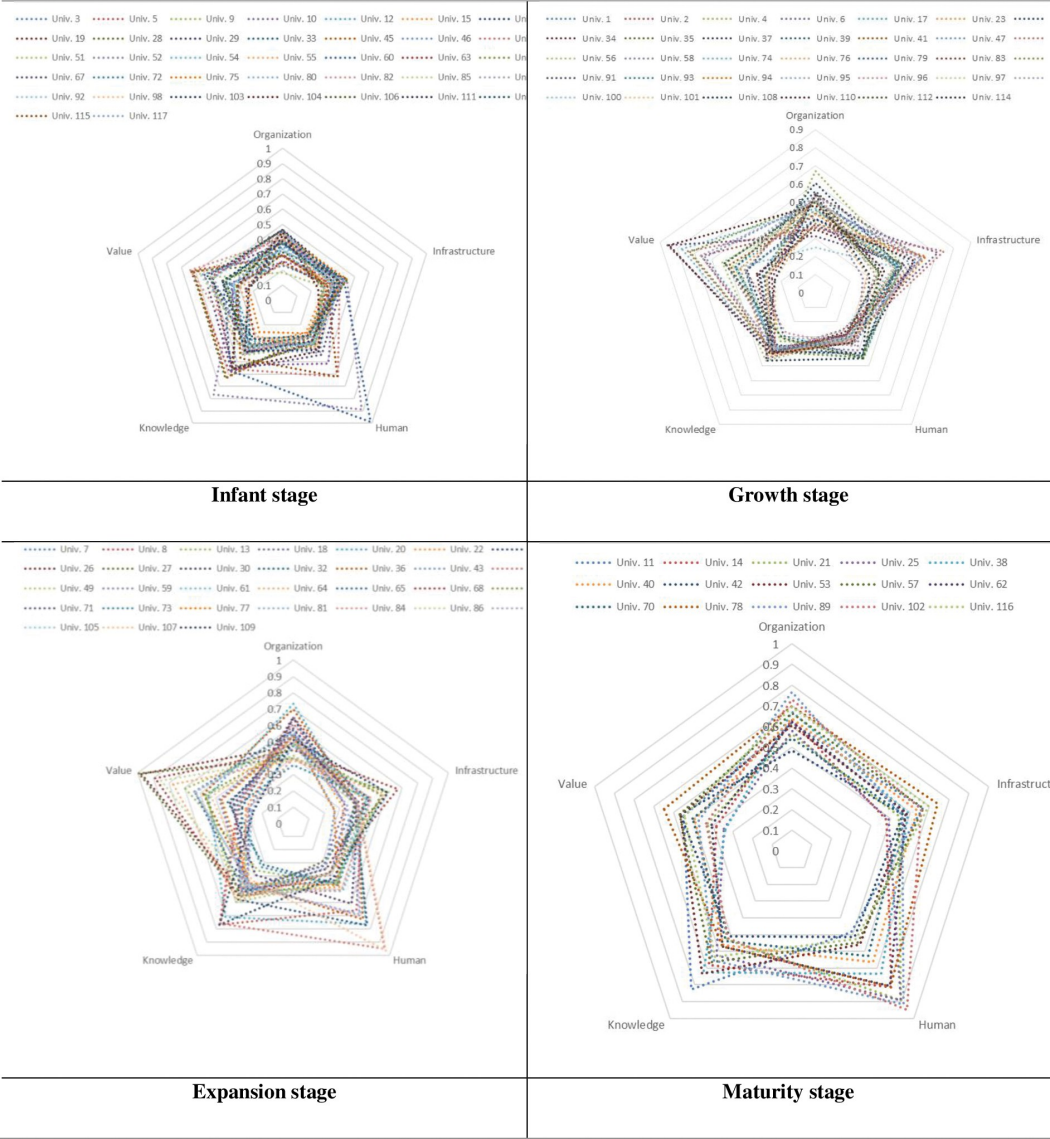
Diagnosis results of technology transfer donors.

To develop a strategic proposal for each stage of the growth of technology transfer providers based on the diagnostic diagram of technology transfers, the previous phase determined which technology transfer provider belonged to which growth stage of technology transfer. Based on this, the Fig 4 were obtained by measuring the technology transfer scores of all the technology donors from five perspectives.

In the case of universities in the infancy stage, most universities showed low scores in institutional perspectives on technology transfer. Therefore, new technology transfer universities should pursue strategies to accelerate technology transfers and move towards the next stage of growth by involving intermediaries, experts, and technology transfer offices. In the case of universities in their growth stage, most showed low scores from the knowledge perspective of technology transfers. Therefore, growing technology transfer universities should pursue strategies to develop technology transfers and move to the next growth stage by increasing their R&D, academic research, technology, and knowledge. In the case of universities in the expansion stage, most showed low scores in the value and organizational perspectives of technology transfers. Therefore, sustainable technology transfer universities should pursue a strategy to evaluate technology transfer efficiency and move to the next growth stage by calculating human and financial resources and developing organizational establishment and culture. In the case of universities in the maturity stage, most showed low scores in the value perspectives of technology transfer. Therefore, advanced technology transfer universities should pursue strategies to evaluate technology transfer efficiency and move to the next growth stage by evaluating technology transfer efficiency and conducting research to further develop technology transfers.

## Section 5: Discussions

To activate technology transfer, various studies are being conducted. Studies related to technology transfer activation can be classified into those that provide activation strategies and those that present technology transfer models. Studies that provide activation strategies can be further classified into those that analyze the establishment of institutions or policies for technology transfer activation, and those that derive and verify success and failure factors. Research examining the dependence between the characteristics of technology donors and the success of technology transfer had been actively being conducted [169]. The research related with the technology donor’s capabilities have been investigated in various studies [168, 251–253]. Additionally, technology transfer models can be classified into theoretical studies that present technology transfer processes, and case studies that validate the presented models. Research had been conducted to evaluate technology transfer at universities, but most research had only reflected a narrow aspect of university technology transfer. As a result, multidimensional evaluations of technology transfer have not been performed, and evaluations have been limited to narrow aspects such as cognitive, graphical, organizational, or social aspects. Furthermore, despite the need to derive optimal strategies for improving the efficiency of technology transfer based on evaluations, previous studies had failed to connect evaluation with strategy derivation.

The growth stage of a technology donor affects the type of technology transfer and the technology transfer strategy. Some researchers have classified the growth stages of technology donors from a technology transfer perspective and suggested appropriate technology transfer types according to the growth stages [216]. However, evaluation in prevision research had been only conducted from a limited organizational perspective. Therefore, this study presents a diagnostic framework for facilitating technology transfer from the perspective of universities, that is, technology donors, and suggests strategies for each growth stage of technology transfer providers to facilitate technology transfers.

The distinctiveness of this study lies in its multidimensional diagnosis and strategy formulation based on the diagnosis. It is able to assess multidimensional perspectives, because CSFs and PMs were extracted based on BSC. Furthermore, by comparing the perspectives score of technology donors in different universities, technology donors can identify the areas in which each university is lacking in its current situation. Multidimensional diagnosis and aggregation score of technology donors offer to extract optimal CSFs for technology transfer activation for each growth stage. Therefore, we compared this study with previous technology transfer evaluation studies based on the criteria of multidimensional diagnosis and strategy formulation. The comparison results are shown in the following Table 8. The following table presents a comparison of this research with previous studies based on the diagnosis target and strategy suggestions. Previous research has focused on technology transfer strategies without evaluating technology transfer activities. Despite the need to comprehensively assess the knowledge and expertise related to technology for successful technology transfers, most research has been developed using evaluation and diagnostic methods to target technology only.

**Table 8:** Comparison of previous research.

	Diagnosis target			Strategy presentation		
Research	Technical and intellectual aspects	Aspects of TTP within the University	Institutional aspects of Universities	Organizational Perspective Strategy	Technology transfer activation strategy	Classification of Growth stages of Universities
[240, 250]	O	X	O	O	X	X
[219]	O	X	O	O	X	X
[137, 169]	O	X	O	O	X	X
[190, 198]	X	O	X	O	X	O
[1, 249]	X	X	X	O	O	X
[241, 242]	O	X	X	O	O	X
[243]	O	X	X	X	O	X
[244–248]	O	X	X	O	X	X
[207]	O	O	O	O	X	X
[129]	O	O	O	X	X	X
[202, 206]	X	O	O	X	X	X
[204]	O	O	X	O	X	X
Our research	O	O	O	O	O	O

## Section 6: Conclusions

### Contributions

The rapid development of technology had created limitations for all companies to develop technology and as a result, the demand for technology transfer was gradually increasing. In Korea, technology transfer is carried out from public research institutes to companies. While technology transfer from government-funded research institutes to companies was relatively active, technology transfer from universities to companies was not insufficient. Many studies had been conducted to remove barriers to technology transfer from universities to companies. However, for successful technology transfer, strategies for selecting technology donors based on the evaluation is necessary. In addition, in order to activate technology transfer from universities, it is necessary for universities to identify their weaknesses as technology donors and to develop strategies for maximizing resource utilization. Therefore, a diagnosis framework for activating technology transfer from the viewpoint of the technology donors rather than the technology users who received the technology was developed. Moreover, the framework was applied to Korean universities.

To provide an accurate diagnosis of technology donors, the study proposed two stages of framework: the first stage is to derive PMs based on the BSC and the second stage is to provide normalized score for each perspective based on normalization and aggregation method. To derive PMs, barriers to technology transfer were identified and strategies to reduce them were developed. CSFs were then derived based on the strategies and verified through a cause and effect relationship. Finally, PMs were derived to measure CSFs. A relative importance weight was extracted using the AHP to derive score for each perspective of technology donors. The normalization and aggregation methods were used to provide a score for each perspective. Finally, universities were classified by growth stage and through relative comparison between universities belonging to each growth stage, lacking perspectives was identified for each university. As technology donors, universities can increase the efficiency of technology transfer by selecting and implementing strategies and CSFs to raise the insufficient perspective score.

To verify the applicability of the research framework presented in this study, we applied the framework to universities in Korea that were playing the role of technology donors. As a result, universities in the maturity stage were found to have high scores in all perspectives. However, Univ.11 had a relatively lower score in the value perspective compared to other universities in the maturity stage. While Univ.11 had a high level of infrastructure, institutional organization, human resource support, and knowledge support, the efficiency and effectiveness of technology transfer were still insufficient. As technology transfer donor, Univ11 needs publicity so that technology transfer can take place. The perspective scores of the 65 universities in the expansion and growth stage showed a significant difference. Among the 65 universities, there were many engineering-oriented universities. Univ.24, Univ.64, Univ.68, and Univ.69, which were engineering-oriented universities, did not have well-established systems and organizations, but technology transfer was relatively active. These universities are expected to have more active technology transfer if they improve their institutions and organizations. The 37 universities in the infant stage had relatively low scores in all perspectives. Therefore, it is necessary to strengthen the TTO for technology transfer by reorganizing the infrastructure and replenishing manpower.

The implications obtained through this study are as follows. First, technology transfer barriers, which are the main factors hindering technology transfer, were redefined. Previous studies that defined technology barriers were unable to comprehensively present technology barriers by only deriving technology barriers from a specific perspective. In this study, barriers to technology transfer reflecting various perspectives were extracted through extensive literature research. Second, most studies related to technology transfer had been focused on technology users, but in this study, it has been focused on technology donors. It is important to select technology donors from the viewpoint of a technology user. However, it is also important to increase the efficiency of technology transfer through various knowledge and support systems of technology donor. By presenting the diagnosis framework based on evaluation of technology donor, it is expected that it will be possible to activate technology transfer through the development of technology donors. Third, CSFs and PMs directly linked to the success of technology transfer were derived. CSFs that were directly related to the success of technology transfer were derived based on the BSC. Through the CSFs, universities can develop strategies to activate technology transfer. Fourth, the aggregation and normalization method that derived the score for each perspective of the university was presented and applied. Since what can be measured can be improved, the score allows universities to identify where they are lacking as technology transfer donors.

### Limitations

This study has some limitations. First, it only focused on domestic universities and conducted evaluations on some of them. There are 143 universities and 138 public research institutes in Korea that provide technology transfer, but due to time and physical limitations, this study only evaluated some domestic universities using the framework derived from this study. A comprehensive survey will be needed to evaluate all technology donors. Second, although evaluations were conducted on technology donors, it was not possible to evaluate whether technology donors who received high scores have actually successfully transferred their technology. Since evaluating technology transfer cannot be done in a short period of time, this study did not carry out such an assessment of technology transfer, but it would be worthwhile to confirm it in the long run in future research. Third, although there are various forms, such as university-company, university-university, and intra-university technology transfer, only technology transfer between universities and corporations was able to diagnosed based on framework of this study. Therefore, the subject of this research was limited solely to university-company technology transfers. Finally, a mathematical method is needed to validate the CSFs of technology transfer. In previous studies, some CSFs had been validated. The effect of individual CSFs on technology transfer had been validated based on statistical method. However, studies on how various CSFs interact with each other to affect technology transfer are lacking. As this study focused on performance evaluation, it was not statistically tested. However, if the CSFs presented in this study is mathematically verified, it will be possible to increase the reliability of the influencing CSFs affecting technology transfer.
